# At-risk and intervention thresholds of occupational stress using a visual analogue scale

**DOI:** 10.1371/journal.pone.0178948

**Published:** 2017-06-06

**Authors:** Frédéric Dutheil, Bruno Pereira, Farès Moustafa, Geraldine Naughton, François-Xavier Lesage, Céline Lambert

**Affiliations:** 1 Université Clermont Auvergne, CNRS, LaPSCo, Physiological and Psychosocial Stress, CHU Clermont-Ferrand, University Hospital of Clermont-Ferrand, Occupational and Preventive Medicine, WittyFit, Clermont-Ferrand, France; 2 Australian Catholic University, Faculty of Health, Melbourne, Victoria, Australia; 3 CHU Clermont-Ferrand, University Hospital of Clermont-Ferrand, the Clinical Research and Innovation Direction, Clermont-Ferrand, France; 4 CHU Clermont-Ferrand, University Hospital of Clermont-Ferrand, Emergency department, Clermont-Ferrand, France; 5 University of Montpellier, Laboratory Epsylon EA 4556, Dynamic of Human Abilities & Health Behaviors, CHU Montpellier, University Hospital of Montpellier, Occupational and Preventive Medicine, Montpellier, France; National Yang-Ming University, TAIWAN

## Abstract

**Background:**

The visual analogue scale (VAS) is widely used in clinical practice by occupational physicians to assess perceived stress in workers. However, a single cut-off (black-or-white decision) inadequately discriminates between workers with and without stress. We explored an innovative statistical approach to distinguish an at-risk population among stressed workers, and to establish a threshold over which an action is urgently required, via the use of two cut-offs.

**Methods:**

Participants were recruited during annual work medical examinations by a random sample of workers from five occupational health centres. We previously proposed a single cut-off of VAS stress in comparison with the Perceived Stress Scale (PSS14). Similar methodology was used in the current study, along with a *gray zone* approach. The lower limit of the gray zone supports sensitivity (“at-risk” threshold; interpreted as requiring closer surveillance) and the upper limit supports specificity (i.e. “intervention” threshold–emergency action required).

**Results:**

We included 500 workers (49.6% males), aged 40±11 years, with a PSS14 score of 3.8±1.4 and a VAS score of 4.0±2.4. Using a receiver operating characteristic curve and the PSS cut-off score of 7.2, the optimal VAS threshold was 6.8 (sensitivity = 0.89, specificity = 0.87). The lower and upper thresholds of the *gray zone* were 5 and 8.2, respectively.

**Conclusions:**

We identified two clinically relevant cut-offs on the VAS of stress: a first cut-off of 5.0 for an at-risk population, and a second cut-off of 8.2 over which an action is urgently required. Future investigations into the relationships between this upper threshold and deleterious events are required.

## Introduction

Workplace stress is a concern for both workers and employers because it can be the cause of absenteeism [1,2,3] and various pathologies such as anxiety [4], depression [4] and cardiovascular diseases [5,6,7]. To fight against work-related stress, and improve occupational health and safety concerns, the European directive on stress at work stated that occupational physicians needed to assess psychosocial risks [8].

Stress can be measured through questionnaires such as the Perceived Stress Scale (PSS) of Cohen [9] or Karasek’s scale [10]. However, their length and complexity render them impractical especially due to the usually limited time of occupational medicine visits [11]. The visual analogue scale (VAS) was originally developed to assess the perception of pain [12]. It has also been used to assess a variety of subjective feelings [13,14,15,16,17,18], including stress [13,14,17,18,19]. The use of VAS is attractive because it is simple to implement, easy to understand by the patient, and time-efficient in execution. Currently, the VAS is the common tool used by occupational physicians to assess stress among workers [13,14,19].

VAS was validated for stress assessment in clinical practice [11,19]. A single cut-off point was proposed for clinical discrimination between stressed and non-stressed workers [11]. However, a single cut-off point (black-or-white decision) inadequately discriminates between workers with and without stress [20]. The most stressed workers are known to commit suicide [21,22,23] and require urgent action. Identifying this group of workers is crucial. Moreover, occupational physicians have limited time to deal with considerable numbers of workers and worksites [24]. As such, physicians cannot thoroughly investigate the large proportion of stressed workers identified by a single threshold. Physicians don’t have time to go to workplaces of every stressed workers to investigate working conditions, to individually clarify the situation with managers, and to find solutions. Identifying workers under the highest pressure with improved accuracy would assist by reducing the numbers in this group to a manageable sample size. Conversely, as stress can vary over time, occupational physicians also need to consider more “potentially stressed” workers who might later need an intervention. “Potentially stressed” workers can be easily followed by the nursing staff sparing time for occupational physicians. Thus, it could be useful to distinguish among workers: (i) a low level of stress not requiring follow-up; (ii) an intermediate zone for further questioning by the clinician; and (iii) a high zone requiring immediate investigation and action. This approach can be modelled with the use of a *gray zone* [20].

Therefore, the aim of this study was to distinguish an at-risk population among stressed workers, and to establish a threshold over which action is urgently required, with the use of two cut-offs comprising the borders of the *gray zone*. The lower limit supports sensitivity (at-risk threshold–under closer surveillance) and the upper limit supports specificity (intervention threshold–emergency action required).

## Methods

### Participants

A previous study from our team proposed a cut-off of stress using VAS in comparison with PSS14 of Cohen [11]. We used the same methodology and we applied the *gray zone* approach. Workers were a random sample of workers from five occupational health centres in four different administrative regions in France. Those occupational health centres followed workers from private companies. Workers were recruited during annual work medical examinations. No consent was given because we used anonymous data from standard clinical records, and only access responses in questionnaires. The ethics committee from the university hospital of Clermont-Ferrand, France, assessed that there was no need for ethics approval.

### Outcomes

Participants were asked to anonymously complete the PSS14 of Cohen [9], and to evaluate their level of stress by moving a cursor on a horizontal, non-calibrated line of 10 cm, ranging from very low (0) on the left to very high (10) on the right [13].

### Statistics

It was difficult to propose sample size estimation according to literature in order to distinguish an at-risk population among stressed workers, and to establish a threshold over which action is urgently required, via the two cut-off points of the *gray zone*. Also, the sample size was limited to the available workers from several occupational health centres in different administrative regions in France. However, a posteriori statistical power calculation was proposed and is further clarified in the discussion.

Statistical analysis was performed using R software (http://cran.r-project.org/) and Stata software, version 13 (StataCorp, College Station, TX, US). All tests were two-sided, with a Type I error set at an alpha level of 0.05. Continuous data were expressed as means and standard deviations (SD) or as medians with interquartile ranges [IQR], and categorical parameters were reported as frequencies and relative percentages. The association between scores on the PSS14 and VAS was assessed with Spearman’s *r* correlation coefficient. First, based on previous data [11], workers were divided into two groups according to a PSS14 score cut-off set at 7.2. Comparisons between independent groups were analysed using the ‘N-1’ Chi-squared test [25] for the categorical variable of gender, and by the Mann-Whitney test for quantitative parameters (age and VAS score), as appropriate. The Gaussian distribution was verified by the Shapiro-Wilk test and homoscedasticity by the Fisher-Snedecor test. Then, a receiver operating characteristic (ROC) curve was used to illustrate the discriminating power of the VAS. A 95% confidence interval (CI) of the area under the ROC curve (AUC) was determined according to the method of DeLong et al. [26]. A “high stress” cut-off for the VAS was then determined by: maximising Youden’s index (J = sensitivity + specificity– 1); maximising the product of sensitivity and specificity [27], and choosing the nearest points to 0, and 1 on the curve. From these three methods, a *gray zone* was used to determine the values of the VAS for which workers were potentially stressed. This approach has been developed as a combination of two methods: bootstrap (sampling with replacement) and Two-Graph ROC (TG-ROC) [28]. The first bootstrap method generates 1,000 bootstrapped samples, determines the best threshold for each sample, and builds a 95% CI of them using their observed distribution (percentile confidence interval). All bootstrapped samples used the same size as the original sample. We used bootstrap in a conventional way (without any conditions) and also bootstrapped samples with the same prevalence of “stressed” workers (those for which PSS14 ≥7.2) as the original sample, in order to check that results were the same. The second method (TG-ROC) consisted of defining three classes of responses: negative, inconclusive, and positive. The inconclusive zone corresponded to the interval of cut-offs for which neither the sensitivity nor the specificity was greater than 0.90; illustrated by a two-curve representation (sensitivity, specificity). These curves were estimated using an artificial neural network. Bootstrap and TG-ROC are complementary, thus the largest interval was defined as the *gray zone*.

## Results

### Participants

**Fig 1:**
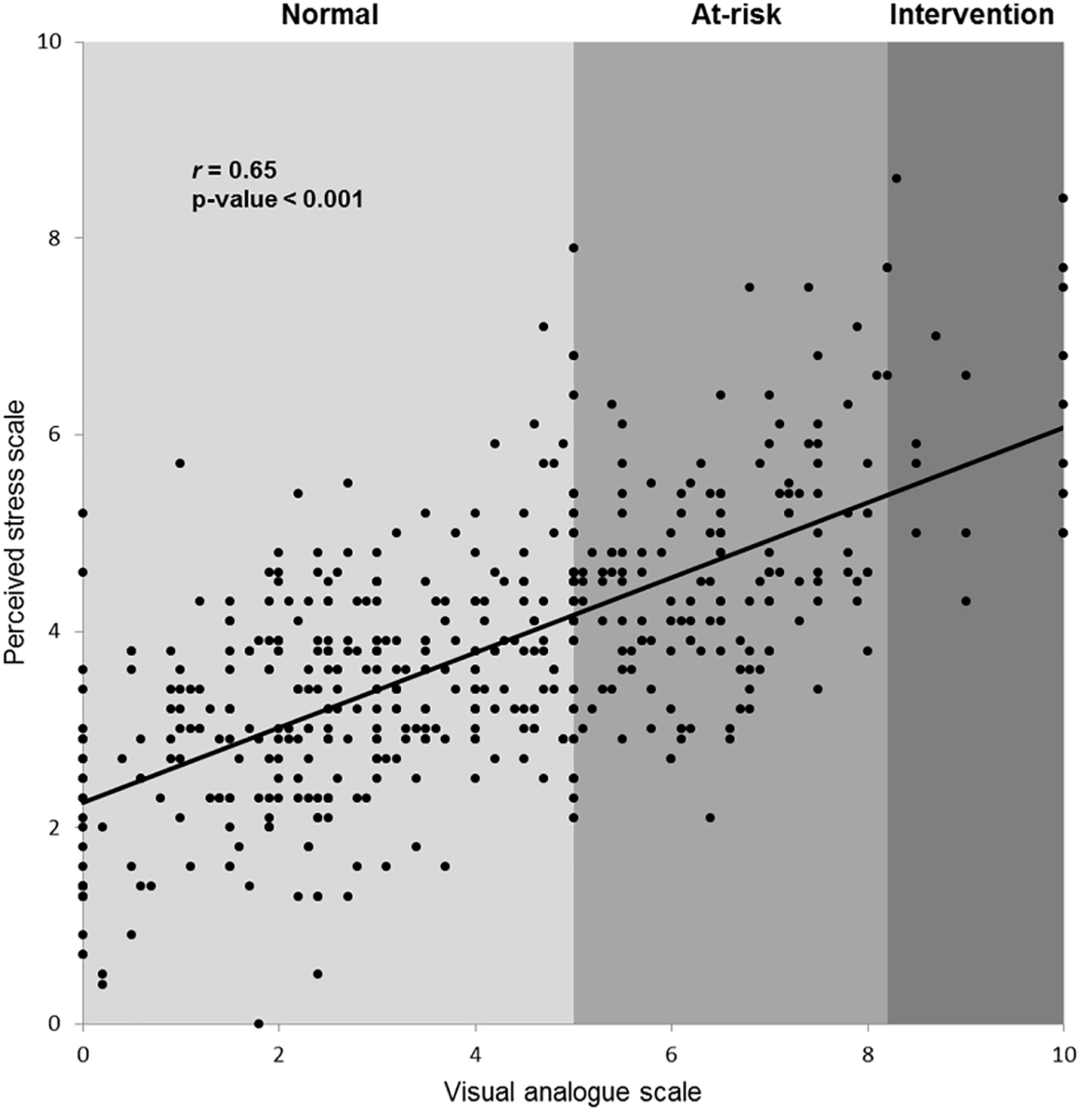
At-risk and intervention thresholds from the relationship between the Perceived Stress Scale (PSS) score and the visual analogue scale (VAS) score.

We tested 500 workers (248 men, 252 women), aged 40±11 years, with a PSS14 score of 3.8±1.4 and a VAS score of 4.0±2.4. PSS14 and VAS were correlated (*r* = 0.65; p<0.001) (Fig 1). Nine (1.8%) of the 500 participants had a PSS14 score ≥7.2. Considering PSS14 as a binary variable (<7.2 vs. ≥7.2), there was a significant difference between gender (50.3% men with a PSS14 <7.2 vs. 11.1% men with a PSS14 ≥7.2, p<0.05) The VAS score differed between those with a PSS14 <7.2 (VAS = 3.8 [2.2–5.5] compared with individuals with a PSS14 ≥7.2, who had a VAS score of 8.2 [7.4–10.0] (p<0.001). In contrast, no significant differences were observed in age, education, professional roles, and occupational sectors (Table 1), as well as for occupations (Supplemental file S1 Database for complete list of occupations).

**Table 1:** Characteristics of workers according to the Perceived Stress Scale considered as a binary variable (<7.2 and ≥7.2).

	PSS14 <7.2 (n = 491)	PSS14 ≥7.2 (n = 9)	p-value
**Gender** (male), n (%)	247 (50.3)	1 (11.1)	<0.05
**Age** (years), median [IQR]	40 [30–50]	46 [41–48]	NS
**VAS of stress**, median [IQR]	3.8 [2.2–5.5]	8.2 [7.4–10.0]	<0.001
**Education**, n (%)			
High-school diploma (US) / A-level (UK) or less	219 (44.6)	5 (55.6)	
Bachelor/undergraduate	174 (35.4)	3 (33.3)	NS
Master degree / postgraduate	98 (20.0)	1 (11.1)	
**Professional roles**, n (%)			
Unskilled workers	171 (34.8)	5 (55.6)	
Skilled workers	109 (22.2)	1 (11.1)	NS
Mid-level workers	136 (27.7)	3 (33.3)	
Senior executives	75 (15.3)	0 (0)	
**Occupational sectors**, n (%)			
Manufacturing/blue collar industries	125 (25.5)	2 (22.2)	
Building industry	11 (2.2)	0 (0)	
Commercial activities and service	123 (25.1)	1 (11.1)	
Agribusiness / food industry	51 (10.4)	1 (11.1)	NS
Transport workers	11 (2.2)	0 (0)	
Hospitality workers	4 (0.8)	0 (0)	
Health and social services	166 (33.8)	5 (55.6)	

PSS14, 14-item Perceived Stress Scale; IQR, interquartile range; NS, not significant; VAS, Visual Analog Scale.

### A single cut-off with ROC-curve approach

**Fig 2:**
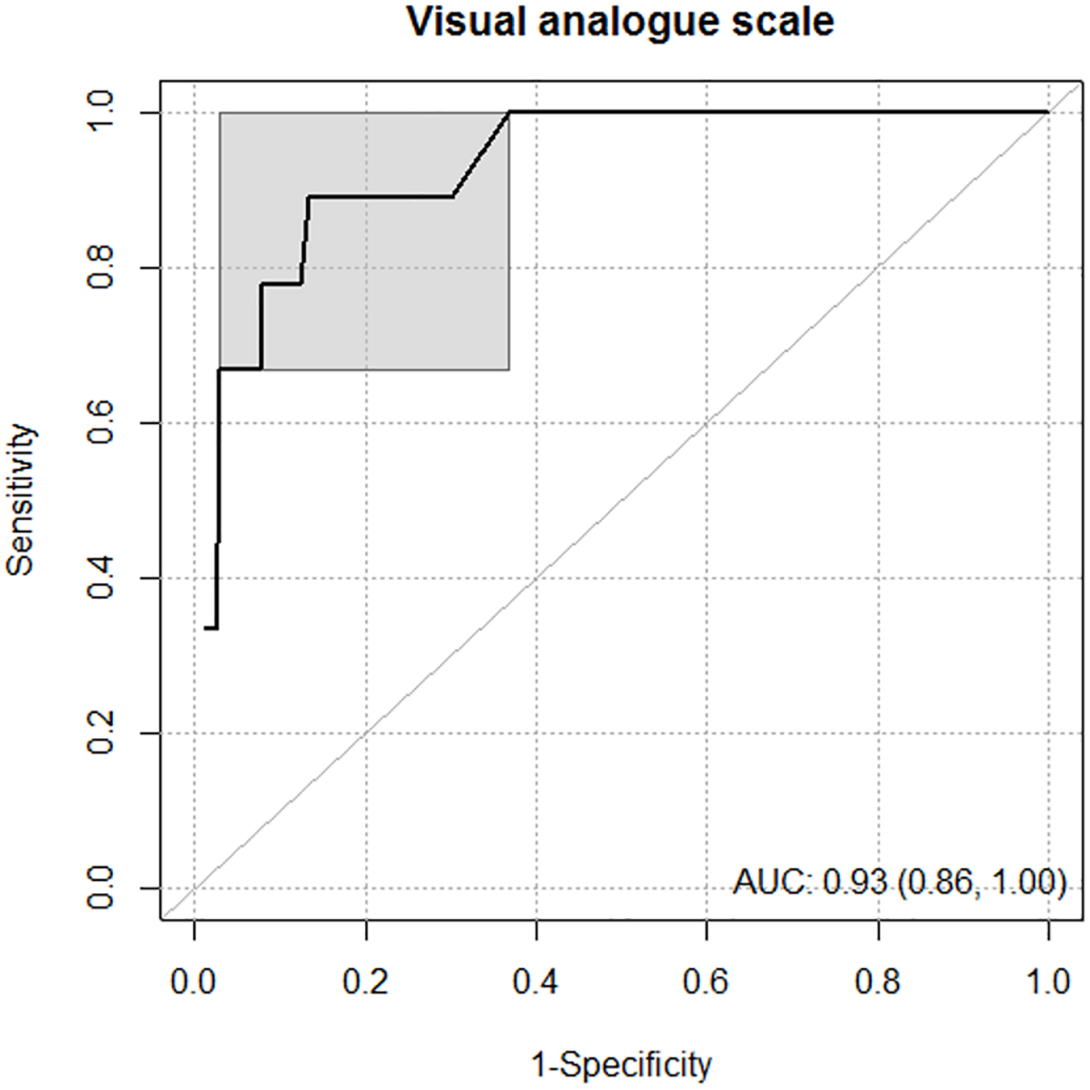
Receiver operating characteristic curve (ROC) representing the discriminative power of the visual analogue scale score to predict a perceived stress scale (PSS14) score greater than, or equal to 7.2. The area under the curve (AUC) is presented with 95% confidence interval. The *gray zone* is represented by the gray square.

Using a ROC curve approach, the global discriminative power of the VAS was significant with an AUC of 0.93 (95% CI: 0.86–1.00) (Fig 2). The optimal VAS threshold was 6.8 (sensitivity = 0.89, 95% CI: 0.57–0.98; specificity = 0.87, 95% CI: 0.83–0.89) independent of the method used (maximising Youden’s index, maximising the product of sensitivity and specificity, and choosing the nearest point to 0, and 1).

### The *gray zone* approach: Determination of at-risk and intervention threshold

**Fig 3:**
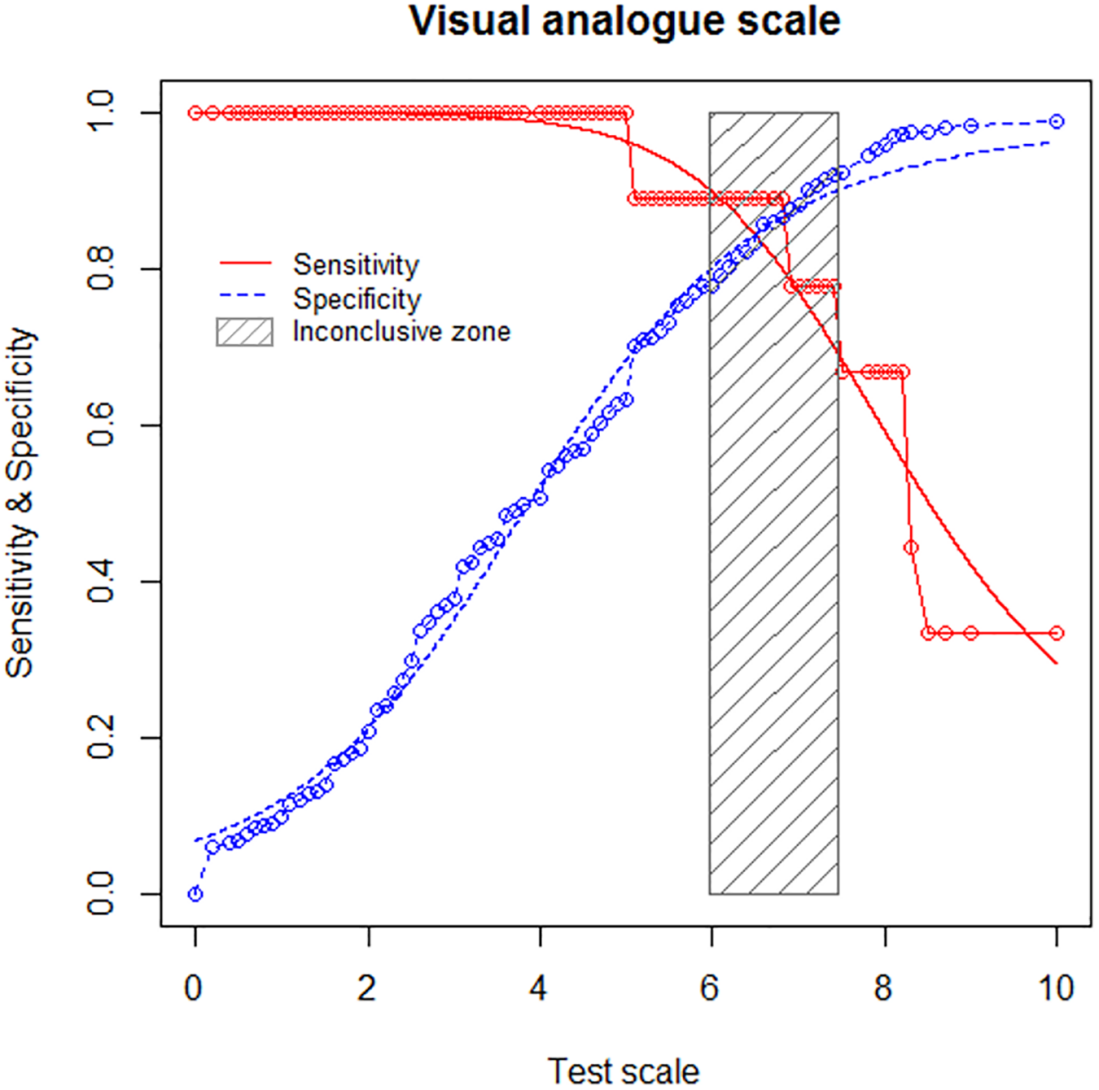
Two-graph receiver operating characteristic curves (TG-ROC): Sensitivity and specificity of the visual analogue scale score according to the perceived stress scale (PSS14) cut-off of 7.2. The inconclusive zone (hatch rectangle) corresponds to the interval of cut-offs for which neither the sensitivity nor the specificity are greater than 0.90.

An optimal threshold was then determined for 1,000 bootstrapped samples having the same prevalence of ‘stressed’ workers as the original sample (*i*.*e*. 1.8%), and the 95% CI of their distribution was 5.0 to 8.2 for each of these three methods. Without requiring any condition to realize sampling with replacement, the 95% CI was exactly the same. Using a TG-ROC, an inconclusive zone ranging from 6.0 to 7.5 was retrieved (Fig 3). The results of bootstrap sampling and TG-ROC were merged into a single *gray zone* (the larger one); 5.0 to 8.2. This *gray zone* included 172 workers, *i*.*e*. 34.4% of the whole sample (at-risk). Seventeen workers of 500 (3.4%) were above the upper limit (intervention). Using the single threshold of 6.8, 74 workers (14.8%) would have been considered as stressed workers. Without the use of a *gray zone* approach, 115 (23.0%) workers potentially at risk, would not have been identified (those with a VAS score between 5 and 6.8).

## Discussion

The main finding of this study was the identification of two cut-offs for stress using the *gray zone* approach, a first cut-off of 5.0 to distinguish an at-risk population among stressed workers, and a second cut-off of 8.2 over which action is urgently required.

### VAS of stress: A discriminant tool

We confirmed with a larger dataset, the previous finding of a relationship between the VAS and the PSS14 of Cohen (*r* = 0.65, p<0.001 in the current study and *r* = 0.68, p<0.001 in a previous sample [11]). Results support the discriminative power of the VAS score to predict a PSS score ≥7.2 [11]. Using three different methods to determine cut-off points, we retrieved an optimal VAS threshold of 6.8 to predict a PSS score ≥7.2, which is close to the threshold of 7.0 previously reported in a smaller sample size [11].

### The *gray zone* approach: A three-zone analysis

Despite confirmation that the VAS is a useful and valid tool for the assessment of stress by occupational physicians, the use of a *gray zone* shows the limitations of using a single cut-off point to support clinical application [20]. Similar to photography, a black and white picture is less accurate than a grey scale one. A three zones analysis is postulated to provide a better compromise between a pragmatic and an accurate assessment. The distinction of three levels (low, moderate, high) is common both in psychological evaluation i.e. Maslach burn-out inventory [29,30,31], and in medical evaluation i.e. probability [32,33] or activity of a disease [34]. For assessing stress, and because occupational physicians may also experience burn-out due to the overload demand [24], they need to differentiate an at-risk population from workers requiring urgent action within the workers previously identified with the highest stress. Indeed, while a single cut-off point previously dichotomized the workers into stressed and unstressed, the *gray zone* provided two thresholds. First, workers with a VAS score lower than the lower limit of the *gray zone* can be confidently considered as unstressed workers. Then, workers with a VAS score in the *gray zone* can be seen as “potentially stressed”. For these individuals, an extended interview can be conducted; preventive action may be implemented and a closer follow-up should be proposed. A non-negligible proportion (23.0%) of workers potentially at risk would not have been identified with the use of a single threshold. This demonstrates another benefit of the use of a *gray zone* approach. Also, workers with a VAS score greater than the upper limit of the *gray zone* can be confidently considered as workers under pressure, for whom action is urgently required. Indeed, the most stressed workers are known to commit suicide [21,22,23].

### Management of workers based on stress levels

As it is impossible for an occupational physician to thoroughly and urgently investigate a vast number of workers, the use of a *gray zone* permitted a reduction in the number of workers in need of urgent action, to a manageable sample, i.e. from 74 workers via the previous single cut-off to 17 via the use of a *gray zone*. Conversely, the number of “potentially stressed” workers became rather large. Nonetheless, the number of workers in the *gray zone* is relatively more easily manageable in occupational medicine. The more advanced interview can be assessed easily by simple questions during routine medical examination. Moreover, preventive action may be implemented by the occupational team. Intervention at the workplace can be conducted by occupational therapist. And the closer follow-up can be easily provided by the nursing staff. Indeed, occupational consultations can be conducted by an occupational physician or by a nurse with specific diploma, based on the decision of the occupational physician for each worker. Naturally, workers requiring urgent action may also benefit from the whole occupational team. Lowering the number of workers above the intervention threshold for whom emergency action is required is crucial because occupational physicians are over-worked and are themselves under pressure [24]. Indeed, occupational physicians have to manage workers requiring an emergency action themselves and cannot delegate this task. For workers above the intervention threshold, physicians must go to workplaces to investigate working conditions and to find solutions with managers for resolving conflicts [35]. Physicians can adapt working conditions [36] or promote a temporary sick leave. In extreme cases, workers can receive an unfit to work diagnosis [36]. Thus, physicians will benefit from access to a tool that is relatively simple to administer; with a potential to identify a smaller number of the most stressed workers. The increasing number of “potentially stressed” workers with the use of the *gray zone* approach is not problematic for occupational physicians because they can delegate the follow-up to the occupational team, especially to occupational nurses for a closer follow-up with dedicated consultations.

### VAS of stress: A first approach strategy

The conceptual construct of stress is complex [37,38]. The PSS14 is composed of several items estimating the level of stress based on objective stressful events, as well as the ability to cope with challenges and personality. [9]. Both the VAS of stress and the PSS14 appear to measure a similar concept of stress as previously demonstrated [11]. However, because of the usually limited time of occupational medicine consultations [24] and because of the variety of problems that an occupational physician must assess [24], a less time-demanding tool may be more efficient; this is one of the advantage of the VAS for assessing stress. Then, further questionnaires can be used in case-specific situations to explore the cause of stress such as the Karasek’s scale [10,39] or the Siegrist questionnaire [40].

### Medical consequences of stress

High levels of stress promote the onset of multiple chronic and acute diseases such as cardiovascular events [6,7]. Thus, the upper limit of stress that we established at 8.2 on the VAS needs to be considered as seriously in need of medical support. In addition to previously cited interventions (investigation at the workplace, discussion with managers, adapting working conditions, sick leave, unfit to work diagnosis), those workers need medical specialists such as psychiatrists, psychologists, or dedicated consultations in specialized occupational centers for bullying at work [35]. From our sample, we retrieved 17 workers (3.4%) needing urgent intervention. This upper limit is the threshold that favours the specificity. Thus, these 17 workers most certainly required medical support. However, some workers in the *gray zone* (n = 172, at-risk) may also require a medical support. We believe that within these at-risk workers, further questions during medical examination could distinguish the workers needing an intervention. In the absence of a cost effective alternative strategy for careful clinical assessment, the use of a *gray zone* with appropriate screening tools may help the clinician to instigate additional medical support. Considering that an occupational physician follows around 3000 workers per year, this upper intervention threshold seems manageable for the physician. We retrieved 38% of workers within both the at-risk and intervention groups, whereas previous studies reported a variable prevalence of job-related distress depending on the assessment tools [14,41,42,43]. However, there are no epidemiological data on severe stress prevalence in workers. Some studies assessed the impact of stress on stress-related diseases with assessment methods other than VAS [1,2,3,4,5,6,7]. To our knowledge, despite the VAS of stress being linked to physiological measures of stress such as inflammatory cytokines [13] or heart rate variability [44] that is linked to life expectancy [45,46,47,48], no studies have investigated levels of stress measured with VAS and disease. Thus, further cohort studies [49] are needed to investigate the relationships between deleterious events and at-risk and intervention thresholds. Additional VAS detected stress measures could further explore relationships between stress and absenteeism and or, work productivity as previously suggested [3,17,36,50].

### Limitations

This study has some limitations. The replacement of a single cut-off point by a *gray zone* may improve the decision-making process of occupational health practitioners. However, clinical examination and occupational physicians’ observations remain essential for assessing repercussions of stress and to support the most appropriate decisions. Also, the low sample size of stressed workers (n = 9) did not allow separate gender analyses in order to investigate a potentially significant relationship between gender and the PSS14 using a binary analysis (<7.2 and ≥7.2).

As outlined in the statistical descriptions, due to difficulties in estimating sample size, a posteriori statistical power calculation was proposed according to results observed for the primary outcome. The AUC-ROC that determined the global discriminative power of the VAS was 0.93, with CI’s lower limit greater than 0.8 (95% CI: 0.86–1.00). Furthermore, with n = 311, n = 172 and n = 17 individuals, the statistical power was greater than 95% in order to show a difference between groups [0 to 5[, [5 to 8.2] and] 8.2 to 10] (respectively, 2.49 ± 1.38 vs. 6.23 ± 0.99 vs. 9.38 ± 0.70, using sampsi and power one-way commands of Stata software considering inflation of type I error due to multiple comparisons). This statistical power seemed satisfactory despite the imbalanced size of groups [51]. Moreover, as indicated by Feise [52], it is necessary to focus not only on statistical significance, but also on the quality of the research within the study and the magnitude of improvement.

Only a modest correlation was observed between the VAS and PSS14 scores (r = 0.65, p<0.001). However, this does not mean that the PSS is capturing something that the VAS is missing. The multi item scales of the PSS were built to provide a lower variance, but the VAS has demonstrated a greater discriminative capacity than PSS [19]. The VAS, and short screening tools in general, are probably more efficient for clinical use [53].

To conduct a *gray zone* analysis using the VAS of stress, we had to know the binary classification (stress–no stress) using the gold standard (PSS14 in our study). Thus, as PSS14 was already the gold standard, we cannot calculate a *gray zone* for PSS14.

Our 500 workers were similar in demographic characteristics to total population from which they were sampled. However, as workers included in this study were only from private companies, further studies should be conducted with a larger sample including non-private sectors to confirm the generalizability of our results to all workers, and to better understand potential associations between stress scores and demographics, employment sectors and current health status or occupational conditions [36,54], as well as with biomarkers of stress [35,48,55,56]. Investigating these relations in non-workers, such as for people living in institutional care, may also have a considerable interest and provide directions for future interventions.

## Conclusion

The use of a VAS for stress assessment is a useful and suitable tool for occupational physicians to estimate stress during clinical examination. To overcome the imperfection of the discrimination caused by the choice of a single threshold, alternative approaches were considered. We identified, with the use of the *gray zone* approach, two clinically relevant cut-offs on the VAS of stress: a first cut-off of 5.0 for an at-risk population, and a second cut-off of 8.2 over which immediate action is strongly recommended. Future investigations into the relationships between this upper threshold and deleterious events are required.

## Supporting information

S1 Database Titles of columns are written without abbreviations. (XLSX)
